# Efficiency of hospitals in COVID-19 era: a case study of an affected country

**DOI:** 10.1186/s12962-024-00549-w

**Published:** 2024-06-11

**Authors:** Anita Hamdollahzadeh, Bahram Nabilou, Hasan Yusefzadeh

**Affiliations:** 1 Department of Health Economics and Management, School of Public Health, Urmia University of Medical Sciences, Urmia, Iran; 2 Social Determinants of Health Research Center, Department of Health Economics and Management, School of Public Health, Urmia University of Medical Sciences, Nazloo Paradise, Sero Road, Urmia, West Azerbaijan 5756116111 Iran

**Keywords:** COVID-19, Hospital revenue, Hospitalization pattern, Orthopedics, General surgery, Urmia

## Abstract

**Background:**

The COVID-19 pandemic has affected all aspects of human life and society and has damaged the global economy. Health systems and hospitals were not exempted from this situation. The performance of hospitals during the COVID-19 pandemic was affected by policies related to the pandemic and other factors. This study aimed to investigate hospital performance indicators such as admissions and revenue.

**Methods:**

The medical records of patients with selected orthopedic and general surgical diseases were studied in two government hospitals in the capital city of Urmia in the second quarter of 2019, with the same period in 2020. Data were extracted based on the number of medical records, including length of stay, hospitalization type, sex, age, insurance, number of deaths, and readmissions from the medical records department. Payment amounts were collected from the revenue department and Hospital Information System. Two performance indicators, two result indicators, and two control indicators were used. Mean disease-specific revenue, total revenue, length of stay, and bed occupancy rate were calculated for both periods. Data were analyzed using SPSS (version 16) and the Mann-Whitney statistical test.

**Results:**

2140 cases were studied in the two disease groups. An increase was observed in the number of hospitalizations and average length of stay during the pandemic. The mean disease-specific revenue in the quarter of 2020 was higher than in 2019. However, total revenue decreased, and the difference in the mean of total revenue was significant for the two years (*P* = 0.00) in teaching center. The number of readmissions remained unchanged throughout in the pandemic. The number of deaths due to general surgery diseases in 2020 compared to the same period in 2019 was associated with a relative increase.

**Conclusions:**

The COVID-19 pandemic increased the slope of health care costs. The analysis of the studied variables as performance, result, and control indicators showed that hospitalization rate, bed occupancy rate, and total revenue followed a similar and decreasing pattern in the selected hospitals during the COVID-19 pandemic. Hospitals should adopt appropriate strategies so that, in conditions identical to the COVID-19 pandemic, their performance is accompanied by proper management of resources, efficiency, and minimal reduction in revenue.

## Background

In December 2019, patients with pneumonia with an unknown microbial agent were identified in Wuhan, China, and reported to the World Health Organization (WHO) in early January 2020. Subsequently, the disease was named “COVD-19 Disease 2019” (COVID-19) [1–3]. Iran was among the first countries to become involved in the COVID-19 pandemic [4]. The latest COVID-19 pandemic statistics indicate 7,611,583 confirmed cases with 146,253 deaths in Iran from January 3, 2020, to May 31, 2023 [5].

The COVID-19pandemic has affected all aspects of human life and society. In addition to changing the speed, structure, and nature of life, it has damaged the global economy [6, 7]. This disease has caused severe pressure on the entire healthcare system and hospitals [8].

Such global crises have a massive impact on people’s lives and health; however, they impose a heavy economic burden on society directly and indirectly. In this regard, the WHO asked all countries to focus their efforts on financing health systems in response to this pandemic. Therefore, it is necessary to investigate the economic consequences of this critical situation and its effects on healthcare organizations, such as hospitals [9, 10].

However, with the increase in people’s awareness of the COVID-19 pandemic and the observance of precautions, the use of healthcare services such as orthopedics and general surgery significantly decreased. Delays in using these services have resulted in increased disease complications and mortality. Some observations and findings showed that the use of healthcare services for elective and emergency conditions has decreased during the pandemic period [11–13].

During the COVID-19 pandemic, patients refused to go to hospitals due to fear of contracting the disease. In this regard, there were delays in visiting the hospitals and an increased proportion of patients receiving antibiotics [14]. Accordingly, during the COVID-19 pandemic, most hospitals worldwide reduced their resources in inpatient and outpatient departments to manage COVID-19 [15]. To limit the risk of contracting covid in hospitals, consultations, routine diagnostic evaluations, and unnecessary procedures were canceled or postponed [16].

In addition, to limit the spread of the disease and increase admission capacity, many hospitals have postponed or canceled elective visits and admissions while closing outpatient departments. These changes threatened the financial ability of hospitals because elective surgeries, especially in orthopedics, are among the most profitable services of hospitals [17].

The Public Health England reported that many patients missed life-saving treatments during the outbreak of Covid-19. In addition, due to the implementation of national regulations used to maintain social distancing to control the virus’s transmission rate, there was a decrease in referrals for orthopedic services [13, 18]. Some studies have reported that the number of general surgeries during the COVID-19 lockdown was significantly reduced [14].

In the United States, efforts have been made to reduce unnecessary clinical visits and diagnostic tests during the COVID-19 disease to protect patients and healthcare workers from exposure to risk [19]. Orthopedic services were also provided in the form of telephone consultations, outpatient care, postoperative evaluations, and rehabilitation. Medicare and Medicaid Services have called for the expansion of telemedicine services [19, 20].

Based on the several studies, COVID-19 does not have the same effect on hospitals. Some studies have reported that COVID-19 has affected the efficiency and reduced the revenues of government hospitals [21–23]. But few results have also been reported that hospitals responded to the pressure during the pandemic, by increasing their efficiency and productivity [24].

To the best of our knowledge, there has been no published research in northwest of Iran on the performance of hospitals during the COVID-19 pandemic, this study was conducted to determine the hospitalization pattern of orthopedic and general surgery services. The results of such research can lead to a better understanding of the impact of this pandemic on the health system, especially hospitals, and provide practical suggestions for planning hospitals in the face of other waves of COVID-19 or other possible pandemics in the future.

## Materials and methods

This descriptive-analytical study was conducted in two government hospitals in Urmia, the capital city of West Azerbaijan Province in Iran, *in the second quarter of 2019 and 2020.* One of the hospitals was the largest referral and teaching hospital in the province, and the second hospital was the only one affiliated with the Social Security Organization (SSO).

The study population included the Medical Records (MRS) of hospitalized patients with orthopedic and general surgery services in the second quarter of 2019 and 2020. Two elective and two emergency diseases with the highest hospitalization rates in both disease groups (orthopedics and general surgery) were considered according to the number of cases in the MRS Unit of the hospitals and interviews with relevant specialists. Then, the names of the diseases and their specific codes were determined using the International Classification of Diseases 10th edition (ICD 10) in the medical records unit. In both years, the census selected all hospitalized cases in the two disease groups. In total, 1386 cases were examined in 2019 and 754 cases were examined in 2020.

Required data, including the name of the disease, MRS number, length of stay, emergency or elective status of the patient, sex, age, and insurance status, were extracted from the patients’ MRS. Revenue data were collected from the Hospital Information System (HIS) in revenue departments. The number of deaths, readmissions, and bed occupancy rates were extracted from the statistics available in the medical record unit for the two periods under investigation. Six indicators, including the Average Length of Stay (ALoS), number of hospitalizations, number of deaths, readmissions, Bed Occupancy Rate (BOR), and revenue, were used in the study.

Owing to the increase in medical tariffs by legal authorities in 2020, officials in the revenue department (*N* = 6) were interviewed to define the amount of adjustment in the monetary value of services. In the interview sessions the major items constituting the bill of the hospitalized patient, such as tariffs of surgery, anesthesia, beds (private and public rooms), and the cost of medicines, were checked. Based on the interview results, the tariff amounts were adjusted to 20%.

### Data treatment

Selected indicators, including ALoS, number of hospitalizations, number of deaths, readmissions, BOR, and revenue, were classified as performance (two first indices ), control ( two later indices), and result (the last two ) indicators, respectively.

Data analysis was performed using SPSS software (version 16), and graphs were drawn using Excel. The frequency and mean of data related to the number of hospitalizations, average length of stay, bed occupancy rate, revenue, number of deaths, and readmission were investigated and compared between the two periods before and during the COVID-19 outbreak. Owing to the non-normality of the data, the Mann-Whitney U test was used to determine the significance of the difference in means. The significance level of the tests were set at *P* ≤ 0.05.

## Results

A total of 2140 cases of selected common diseases were reviewed before and during the COVID-19 pandemic. One Thousand six hundred forty-eight cases were related to the medical referral teaching center affiliated with Urmia University of Medical Sciences. The number of patients admitted as an emergency was higher than that of elective cases.

**Table 1:** Frequency distribution and percentage of hospitalized patients before and during COVID-19based on demographic and background information

No	Variable		2019		2020		Increase or decrease (%)
			*N*	%	*N*	%	
1.	Sex	Male	850	61.32	504	67.24	− 40.4
		Female	536	38.68	247	32.76	− 54
2.	Age groupe	< 20	208	15	130	17.24	− 37.5
		20–29	229	16.52	121	16.04	− 47.2
		30–39	268	19.33	147	19.49	− 45.2
		40–49	216	15.58	110	14.58	− 49
		50–59	196	14.14	104	13.79	− 47
		> 60	269	19.43	142	18.86	− 47.3
3.	Hospitalization type	Elective	708	51.08	348	46.15	− 51
		Emergency	674	48.92	406	53.85	− 45.5
4.	Insurance coverage	Yes	1029	74.24	529	70.15	− 48.6
		No	357	25.76	225	29.85	− 37

Based on the demographic and background characteristics, hospitalizations for the studied diseases decreased in 2020 compared to 2019. This decrease was higher in males than in females (Table 1).

**Table 2:** Frequency distribution of the number of hospitalizations and percentage changes of selected diseases before and during covid-19

No	Deseases groups		Emergency/ Elective	Codes of Deseases	Year (*N*)		Increase or decrease (%)
					2019	2020	
1.	General Surgery		Emergency	T07	336	187	44.35-
2.			Emergency	K35.8	155	99	36.13-
3.			Elective	K80.2	288	129	55.21-
4.			Elective	K40.9	277	109	60.65-
5.	Ortopeadics		Emergency	S61.0	77	75	2.6-
6.			Emergency	S52.50	106	73	31.14-
7.			Elective	Z47.0	98	47	52.05-
8.			Elective	S72.00	49	35	28.58-
		**Total**			1386	754	45.6-

The total number of hospitalizations for the studied diseases in 2020 decreased by 45.6%. The maximum decrease was observed in the group with general surgery hospitalizations. An emergency-elective comparison revealed the most significant decrease in the number of elective hospitalizations (Table 2).

**Table 3:** Frequency distribution and the average length of stay of selected diseases before and during covid-19

NO	Desease Code	ALoS (standard deviation)		Decrease or increase in 2020 VS 2019	*P*
		The second quarter of 2019	The second quarter of 2020		
1.	T07	3.74 (3.72)	5.16 (4.71)	+ 1.42	≤ 0.001
2.	K35.8	2.37 (1.3)	2.84 (2.1)	+ 0.47	0.055
3.	K80.2	3.87 (2.56)	4.28 (2.6)	+ 0.41	0.067
4.	K40.9	2.12 (1.01)	2.37 (1.03)	+ 0.25	0.011
5.	S61.0	1.21 (0.43)	2.17 (2.86)	+ 0.96	≤ 0.001
6.	S52.50	2.53 (1.92)	2.89 (2.37)	+ 0.36	0.097
7.	Z47.0	2.21 (1.62)	2.3 (1.17)	+ 0.09	0.265
8.	S72.00	5.65 (4.1)	5.77 (5.65)	+ 0.12	0.24

The ALoS of the studied diseases increased during the pandemic. The Mann-Whitney test showed that the difference between the ALoS in the majority of the selected emergency diseases was significant in the two periods (Table 3).

**Table 4:** The percentage of decrease or increase in the mean of Diseases-specific revenue before and during COVID-19

No	Diseases codes		2019 (*N*)	2020 (*N*)	Increase or decrease (%)
1.	General Surgery	T07	336	187	+ 81
2.		K35.8	155	99	+ 12
3.		K80.2	288	129	+ 13
4.		K40.9	277	109	+ 7
5.	Ortopeadics	S61.0	77	75	+ 33.5
6.		S52.50	106	73	+ 13
7.		Z47.0	98	47	+ 8
8.		S72.00	49	35	+ 4
9.	Total		1386	754	+ 38

The mean revenue of selected diseases increased in the general surgery (32.22%) and orthopedic (40.4%) groups, and there was a 38% increase in the total mean revenue in 2020 compared to 2019. The maximum increase in revenue was related to emergency hospitalizations (Table 4).

As service quality indicators, readmissions and deaths of selected diseases investigated in two study periods (Table 5).

**Table 5:** Frequency and percentage of readmissions and deaths compared to total hospitalizations during study periods

NO	Diseases’ Code	2019			2020		
		Admissions	Readmissions (*N* / %)	Deaths (*N* / %)	Admissions	Readmissions (*N* / %)	Deaths (*N* / %)
1.	T07	336	5 (1.5)	13(3.86)	187	10 (5.3)	13 (6.95)
2.	K35.8	155	4 (2.6)	0(0.0)	99	1 [1]	1 [1]
3.	K80.2	288	21(7.3)	1 (0.34)	129	10 (7.75)	2 (1.55)
4.	K40.9	277	5(1.8)	0(0.0)	109	1(0.9)	0(0.0)
**General surgery**			35(3.31)	14(1.32)		22(4.19)	16(3.05)
5.	S61.0	77	1(1.3)	0(0.0)	75	1(1.33)	0(0.0)
6.	S52.50	106	0 (0.0)	2(1.88)	73	0 (0.0)	0(0.0)
7.	Z47.0	98	2 (2.04)	0(0.0)	47	1(2.12)	0(0.0)
8.	S72.00	49	1(2.04)	2(4.08)	35	0(0.0)	0(0.0)
**Ortopedic**			4(1.21)	4(1.21)		2(0.87)	0(0.0)
Total		1386	39 (2.8)	18 (1.3)	754	24 (3.18)	16 (2.1)

In general, the percentage of readmissions and deaths increased in pandemic period, but But comparasion of these indicators based on the two groups of general surgery and orthopedic diseases showed that they were reduced in orthopedic diseases (Table 5).

In the teaching center, the total revenue of selected diseases in the general surgery group faced a greater decrease than that of selected orthopedic diseases, and the total revenue decreased. The Mann-Whitney test showed that the difference in the mean of total revenue was significant (*p* = 0.00) in this hospital, and it was lower in 2019.

In the SSO-affiliated hospital, the total revenue of selected diseases of general surgery faced a greater decrease than that of selected orthopedic diseases, and a greater decrease in revenue was observed overall. The Mann-Whitney test showed that the difference in the average total revenue in this hospital in 2019 was lower, but not statistically significant (*p* = 0.09).

## Discussion

The reduction in the number of hospitalizations and revenue during the pandemic were the most important findings. The ALoS for the studied diseases was more in the 2020. The BOR decreased in 2020 compared to that in 2019. The total revenue of hospitals in 2020 was lower than that in 2019.

The increase or decrease in the performance indicators in the investigated hospitals can be caused by the non-referral of patients, the plans of the hospitals and decisions of doctors. Population reactions and healthcare system responses are essential in the pandemic regarding reduced demand for care and the system’s capability to intercept it [25].

This pandemic was a global challenge for society and healthcare systems, affecting sectors such as surgery and orthopedics owing to nationwide restrictions and restructuring of the healthcare system.

In a study by Haffer et al. (2020), with the cancellation of elective surgeries and hospitalizations in March 2020, the orthopedic admission capacity decreased by 49.4% and the hospital’s revenue reduced by 29.3% [26]. These results are in line with the present study, in which a reduction in the number of hospitalizations caused a decrease in the hospital’s revenues.

In 2020, Wong and Cheung (2020) reported that a reduction in the number of elective surgeries caused a 58.9% decrease in the number of hospitalizations [27]. In another study in 2020, the overall orthopedic activity became more limited compared with the pre-COVID-19 era. Regarding the volume and nature of surgeries performed during the pandemic period, almost complete disruption of elective surgeries has been reported [28]. These studies are consistent with the current study in terms of reducing the number of emergency and elective hospitalizations.

In a study by Murphy et al. in 2020, there was a significant decrease in orthopedic department patients during the COVID-19 pandemic due to reasons such as the suspension of social activities and people’s fear of contracting the disease in the hospital. So, the number of surgeries performed during the pandemic has significantly decreased [13].

In a study conducted by Callan et al. in 2020, the ALoS of general surgery patients increased during the two-week period after the announcement of mandatory quarantine (5.7 vs. 2.3 days). An increase in the ALoS, especially in patients who stayed for 10 days or more, was observed among patients hospitalized during the COVID-19 pandemic. Recent data from the United Kingdom showed a higher-than-expected increase in mortality when considering deaths related to COVID-19 [29].

In the Patels study in 2020, fewer patients were observed during the pandemic than before, and the number of patients and the length of hospitalization decreased significantly, which was accompanied by a delay in going to the hospital. The rate of patient admission by the general surgery team before quarantine was 151, and during quarantine, there were 75 cases, which was equivalent to a 50.3% reduction [14]. In the present study, there was an average decrease of 28.59% in the number of general surgeries performed during the pandemic. There were no significant differences in age or sex between the two groups.

In a study conducted in 2020, 53.4% of the patients were male and 60% were aged > 60 years. Daily admission rates for these acute medical conditions were lower during the COVID-19 pandemic [30].

A 2020 study found that canceling elective surgeries during the COVID-19 pandemic resulted in losses of $16.3 to $17.7 billion per month in reimbursements and $4.5 to $4.5 billion per month in revenue for the US hospital system [31]. The overall revenue of the hospitals selected in the current study was also reduced. An important issue is predicting a decrease or increase in revenue and its amount, which should be done in advance by policymakers and managers of the health system, and countermeasures should be foreseen.

Based the studies conducted in Iran, COVID-19 has affected the efficiency and revenues of government hospitals negatively, which have faced many problems owing to high costs. The revenues of government hospitals were continuously decreased for several months [21]. Mahmoodpour-Azari reported decrease in the BOR and bed turnover rate after the outbreak of COVID-19 and In contrast, increase in the ALoS and bed turnover interval [23]. Kazempour-Dizajialso reported lesser revenue in the period of COVID 19 outbreak [22].

One of the reasons for this decline was the cancellation of elective surgeries and the reduction of hospital visits caused by COVID-19 [32].

In different ountries, the government took action to help hospitals, based on studies showing that the pandemic was likely to weaken the position of hospitals. The financial situation of hospitals before the outbreak of COVID-19 was an important determinant of their ability to neutralize external financial shocks. The devastating impact of COVID-19 showed that some for-profit hospitals, in particular, did not had financial resilience during this crisis. The pandemic may led to significant changes in both for-profit hospitals and healthcare systems (Kruse & Jeurissen 2020). The issue of financial resilience in the hospitals selected in this study was not very prominent because of the availability of government budgets; however, the issue of financial management is one of the main duties of hospital trustees.

## Conclusion

The COVID-19 pandemic increased the upward slope of healthcare costs and showed that selected hospitals were not ready to deal with the pandemic. Examination and comparison of the investigated variables showed that the hospitalization rate, BOR, and total revenue followed a similar and decreasing pattern in the studied hospitals during the COVID-19 pandemic. The control indicators showed that, while the performance of both hospitals dropped, the quality of services dropped.

Based on high-level policies and strategies (which should exist) of the health system, during the pandemic of Covid-19 or other similar conditions, hospitals should adopt appropriate and unique plans so that their performance is accompanied by quality and reasonable income.

### Limitations of the study

In this study, only government hospitals were examined; therefore, the application of the results cannot be generalized to all hospitals and is also limited to general surgery and orthopedic departments.

Regional differences in the spread of COVID-19 may lead to distortions in the assessment because different regions in the country have been affected by the pandemic to different degrees. Also, a relatively short period of time was defined for the evaluation, but it is possible that in the long term, hospitals and the health system will adapt to the conditions, and the results will change.

## Data Availability

No datasets were generated or analysed during the current study.

## Abbreviations

ALoS: Average Length of Stay
BOR: Bed Occupancy Rate
ICD 10: International Classification of Diseases 10th edition
MRS: Medical Records
SSO: Social Security Organization
UMSU: Urmia Medical Science University
WHO: World Health Organization
